# Supplementary material for: Gap junction-mediated glycinergic inhibition ensures precise temporal patterning in vocal behavior
Source: eLife. 2021 Mar 15;10:e59390. doi: 10.7554/eLife.59390 (PMC7963477; doi:10.7554/eLife.59390)
Supplement: Transparent reporting form [file elife-59390-transrepform.docx]

***Life’s* transparent reporting form**

We encourage authors to provide detailed information *within their submission* to facilitate the interpretation and replication of experiments. Authors can upload supporting documentation to indicate the use of appropriate reporting guidelines for health-related research (see [EQUATOR Network](http://www.equator-network.org/%20)), life science research (see the [BioSharing Information Resource](https://biosharing.org/)), or the [ARRIVE guidelines](http://www.plosbiology.org/article/info:doi/10.1371/journal.pbio.1000412) for reporting work involving animal research. Where applicable, authors should refer to any relevant reporting standards documents in this form.

If you have any questions, please consult our Journal Policies and/or contact us: [editorial@elifesciences.org](mailto:editorial@elifesciences.org).

**Sample-size estimation**

- You should state whether an appropriate sample size was computed when the study was being designed
- You should state the statistical method of sample size computation and any required assumptions
- If no explicit power analysis was used, you should describe how you decided what sample (replicate) size (number) to use.

Please outline where this information can be found within the submission (e.g., sections or figure legends), or explain why this information doesn’t apply to your submission:

Our sample sizes are based on prior extensive quantitative studies in another closely related species, within the same brain region (e.g., see Chagnaud et al., 2011, Nature Communications and Chagnaud et al., 2012, Journal of Neurophysiology cited in manuscript). While it may be statistically desirable to use only one neuron from a single fish and repeat the experiment seven times, for ethical reasons to minimize animal number (wild population of animals), we record from multiple neurons from at least two individuals in each analysis. Issue of possible pseudo-replication arising from multiple neurons from the same fish are handled by using nested mixed models. Sample size (number of neurons and fish) are provided for each experiment at the location they are discussed (and quantitatively analyzed) in the result section of the manuscript. We refer to the total amount of animals used in the study in the M&M section.

**Replicates**

- You should report how often each experiment was performed
- You should include a definition of biological versus technical replication
- The data obtained should be provided and sufficient information should be provided to indicate the number of independent biological and/or technical replicates
- If you encountered any outliers, you should describe how these were handled
- Criteria for exclusion/inclusion of data should be clearly stated
- High-throughput sequence data should be uploaded before submission, with a private link for reviewers provided (these are available from both GEO and ArrayExpress)

Please outline where this information can be found within the submission (e.g., sections or figure legends), or explain why this information doesn’t apply to your submission:

We refer to the number of replicates in the manuscript, and we always refer to biological replicates (neurons and fishes). The exact number of neurons used per fish is indicated in supplementary table 1. In our electrophysiological experiments, we were sometimes able to record from several motoneurons in a single fish. In any case, replicates from additional fishes were always performed and added to the analysis. Replicates are reported in the Results section.

**Statistical reporting**

- Statistical analysis methods should be described and justified
- Raw data should be presented in figures whenever informative to do so (typically when N per group is less than 10)
- For each experiment, you should identify the statistical tests used, exact values of N, definitions of center, methods of multiple test correction, and dispersion and precision measures (e.g., mean, median, SD, SEM, confidence intervals; and, for the major substantive results, a measure of effect size (e.g., Pearson's r, Cohen's d)
- Report exact p-values wherever possible alongside the summary statistics and 95% confidence intervals. These should be reported for all key questions and not only when the p-value is less than 0.05.

Please outline where this information can be found within the submission (e.g., sections or figure legends), or explain why this information doesn’t apply to your submission:

In all figures, we show a raw recording file and the sum of the analyzed data is provide where possible as a violin plot. The statistical test used is provided in the materials and method section.

(For large datasets, or papers with a very large number of statistical tests, you may upload a single table file with tests, Ns, etc., with reference to sections in the manuscript.)

**Group allocation**

- Indicate how samples were allocated into experimental groups (in the case of clinical studies, please specify allocation to treatment method); if randomization was used, please also state if restricted randomization was applied
- Indicate if masking was used during group allocation, data collection and/or data analysis

Please outline where this information can be found within the submission (e.g., sections or figure legends), or explain why this information doesn’t apply to your submission:

This does not apply to the experimental design of our study.

Individual neurons from different fishes were used to test the different experimental manipulations.

**Additional data files (“source data”)**

- We encourage you to upload relevant additional data files, such as numerical data that are represented as a graph in a figure, or as a summary table
- Where provided, these should be in the most useful format, and they can be uploaded as “Source data” files linked to a main figure or table
- Include model definition files including the full list of parameters used
- Include code used for data analysis (e.g., R, MatLab)
- Avoid stating that data files are “available upon request”

Please indicate the figures or tables for which source data files have been provided:

We uploaded *.xls files with the data used to generate the plots in figures. We in addition uploaded two additional movies that show a series of original traces showing the described network induced hyperpolarization. The original data has been uploaded to a data repository system: https://doi.org/10.7910/DVN/RZLWHE
